# Supplementary material for: Prolonged Gel Delivery to Oral Cavity from a Silicone Tube: In Vivo Assessment
Source: Pharmaceutics. 2025 Aug 22;17(9):1095. doi: 10.3390/pharmaceutics17091095 (PMC12473675; doi:10.3390/pharmaceutics17091095)
Supplement: Supplementary file 1 [file pharmaceutics-17-01095-s001.zip › pharmaceutics-3792634 - Supplementary material.pdf]

Prolonged Gel Delivery to Oral Cavity from a Silicone Tube: In Vivo Assessment

Supplementary Material Section S1

In vitro and in vivo erosion of hydrogels from silicone perforated tubes – comparison of visual and digital method

Quantitative analysis of erosion occurring both in vivo and in vitro was assessed visually by measuring the length of tube segments not stained blue (Fig. 4). A digital evaluation of the photos using an image analysis program (open source ImageJ program) was also applied, yielding very similar results.

Table: Subject and formulation codes, sampling time points (min), photographic records, and calculated erosion percentages based on manuscript methodology and ImageJ software analysis

Subject and formulation codes	Time point (min)	Photo	Erosion (%)	
			Visual methodology (results analysed in the article)	Image J software
B.J_T1_HEC	60	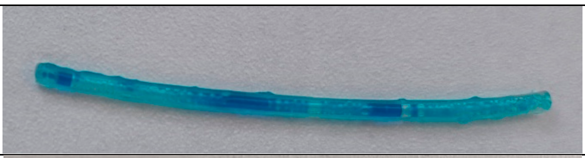	75	77
B.J_T1_C	240	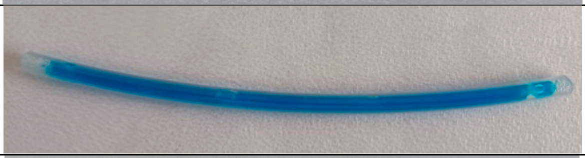	12	13.5
E.W_T1_HEC	150	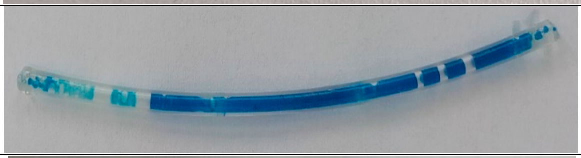	32.5	31
K.P_T1_HPMC	210	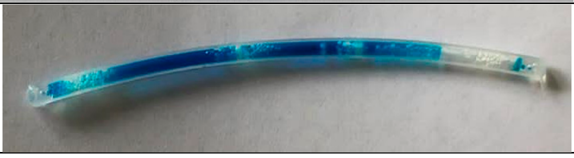	45	48

M.B_T2_HPMC	30	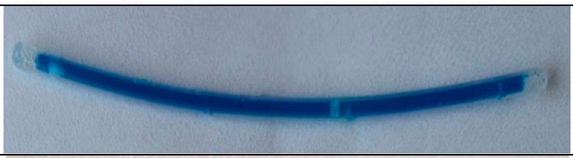	5	5
M.L_T2_HEC	120	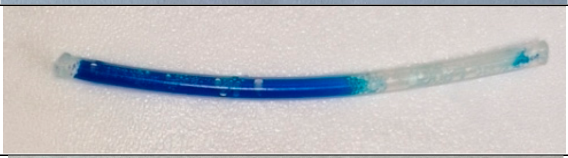	40	41.5
M.P_T1_HPMC	240	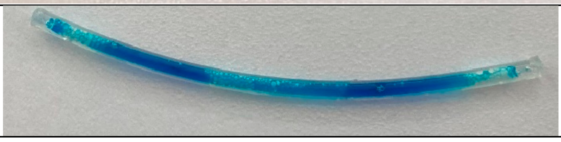	60	60

Other results (in different time points)

Formulation code and subject code	Visual estimation	Image J
BJ_T1_C	12	13.5
BJ_T2_C	5	8.2
BJ_T1_HEC	100	100
EW_T1_C	2.5	3.4
EW_T1_HEC	40	41.5
EW_T2_HEC	20	20
KP_ T1_C	2.5	2.5
KP_T1_HPMC	50	47.5
KP_T1_HEC	45	41
MP_T1_HPMC	30	28
MP_T1_HEC	75	72.5
MP_T2_HPMC	40	45
ML_T2_C	5	5
ML_T2_HPMC	40	35
ML_T2_HEC	90	90
MP_T1_HPMC	60	60
MP_T2_C	5	5
MP_T2_HEC	30	35
AS_T1_HEC	45	47.5
MP_T1_HEC	25	22.5
ZW_T1_HEC	100	100
SA_T1_HEC	70	70

Supplementary Material Section S2

Statistical analysis of in vivo erosion (%) results – confidence intervals (ad Fig. 5)

T1-C: 5.67 (95%, CI: -7.95, 19.29)
T1-HEC: 60.71 (95%, CI: 33.04, 88.39)
T1-HPMC: 46.67 (95%, CI: 8.72, 84.61)
T2-C: 5, N/A (no variation in data)
T2-HEC: 61.67 (95%, CI: -29.89, 153.22)
T2-HPMC: 36.67 (95%, CI: 22.32, 51.0)
